# Lipid Metabolism Affects Fetal Fraction and Screen Failures in Non-invasive Prenatal Testing

**DOI:** 10.3389/fmed.2021.811385

**Published:** 2022-01-12

**Authors:** Jun Cao, Longwei Qiao, Jieyu Jin, Sheng Zhang, Ping Chen, Haoyu Tang, Zheng Yu, Jingye Shi, Ting Wang, Yuting Liang

**Affiliations:** ^1^Center for Clinical Laboratory, The First Affiliated Hospital of Soochow University, Suzhou, China; ^2^School of Gusu, The Affiliated Suzhou Hospital of Nanjing Medical University, Suzhou Municipal Hospital, Nanjing Medical University, Suzhou, China; ^3^Department of Laboratory Medicine, Shanghai General Hospital, Shanghai Jiao Tong University School of Medicine, Shanghai, China

**Keywords:** non-invasive prenatal testing, fetal fraction, cell-free DNA, triglyceride, multivariable linear regression models

## Abstract

**Objective:** To assess the association between lipid metabolism and fetal fraction, which is a critical factor in ensuring a highly accurate non-invasive prenatal testing (NIPT), and on the rate of screen failures or “no calls” in NIPT.

**Methods:** A total of 4,514 pregnant women at 12–26 weeks of gestation underwent NIPT sequencing and serum lipid measurements. Univariate analysis and multivariate regression models were used to evaluate the associations of serum lipid concentrations with the fetal fraction and the rate of screen failures.

**Results:** The fetal fraction decreased with increased low-density lipoprotein cholesterol and triglyceride (TG) levels, which were significant factors (standardized coefficient: −0.11). Conversely, high-density lipoprotein cholesterol and the interval between the two tests were positively correlated with the fetal fraction. The median fetal fraction was 10.88% (interquartile range, 8.28–13.89%) and this decreased with TG from 11.56% at ≤1.10 mmol/L to 9.51% at >2.30 mmol/L. Meanwhile, multivariate logistic regression analysis revealed that increased TG levels were independently associated with the risk of screen failures. The rate of screen failures showed an increase with TG levels from 1.20% at ≤1.70 mmol/L to 2.41% at >2.30 mmol/L.

**Conclusions:** The fetal fraction and the rate of screen failures in NIPT are affected by TG levels. Meanwhile, in pregnant women with high TG levels, delaying the time between NIPT blood collections can significantly increase the fetal fraction.

## Introduction

Massively parallel sequencing of cell-free DNA (cfDNA) in maternal blood is a non-invasive prenatal testing (NIPT) method that has been applied in screening for trisomy 21, 18, and 13 (1, 2). In trisomic pregnancies, cfDNA derived from the extra fetal chromosome leads to a higher proportion of fetal DNA than in disomic pregnancies (3). Indeed, the proportional increase caused by the trisomy chromosome is equal to half the fetal fraction (4). Thus, the accurate identification of fetal aneuploidy depends largely on the detection of the fetal fraction using the NIPT method (5, 6).

The American College of Medical Genetics and Genomics defines low fetal fractions as less than 4% (<4%) (7), which would result in test failures and false-negative results (8). This is assuming that NIPT screening has a 100% trisomy 21 (T21) detection rate at different failure rates. When the failure rate is 1, 5, or 10%, the corresponding detection rate of the screening population declines to 99, 95, and 90%, respectively, assuming that the incidence of T21 among failures is the same as that reported in the literature (9). Indeed, recent evidence suggests that a 99.7% detection rate for T21 (357 of 358) was observed in a study of 58,048 pregnant women with whole-genome sequencing-based NIPT. There were 13 T21 cases with a fetal fraction of <4%. The sensitivities for T21 decreased from 99.7 to 96.4% when considering screening failure cases (10, 11). Therefore, screening failure will negatively influence NIPT performance, and it is recommended to find a variety of other strategies to reduce this rate.

Researchers have mainly focused on two aspects to reduce the rate of screen failures. First, Lo et al. performed plasma DNA size analysis and found that the most significant difference between fetal and maternal cfDNA was a decrease in the 166-bp peak relative to the 143-bp peak (12, 13). From this, they have developed a new NIPT method to enrich shorter cfDNA fragments (<140 bp) to significantly improve the fetal fraction (by 2.3-fold) and analytical performance of NIPT (11, 14–17). However, this new method has not been applied in clinical practice. Second, other researchers have studied the effects of maternal and fetal characteristics and experimental factors on fetal fraction with the aim of adjusting these factors to obtain higher numbers of fetal-derived cfDNA. Fetal fraction can be influenced by maternal body mass index (BMI), gestational age (GA), anticoagulation therapy, blood collection, and fetal aneuploidy (14, 18, 19). Based on prior research, one potential way to reduce the incidence of screening failure is to repeat test failures by sequencing a second sample. The second sample can either be collected in conjunction with the first sample or from a redraw after initial failure due to a low fetal fraction (20). However, recent studies have shown that only 56–66% of repeated NIPT after a second blood draw are successful, suggesting that other factors may also affect the fetal fraction. Serum lipid levels, the main source of cfDNA in the plasma of pregnant women, are extensively associated with peripheral leukocyte counts, and this may affect the fetal fraction.

The aims of this study on 4,514 pregnant women undergoing NIPT at 12–26 weeks of gestation were, first, to examine the possible effects of lipid metabolism on the fetal fraction and second, to estimate the proportion of pregnancies at a high risk for screen failure.

## Materials and Methods

### Study Population

This was a single-center, retrospective study investigating pregnant women who underwent NIPT to screen for trisomy 21, 18, 13 and serum lipid measurement at 12–26 weeks of gestation. Data on 4,514 pregnancies with male fetuses were collected between January 2016 and December 2020 at the Suzhou Municipal Hospital. The study was approved by the Reproductive Medicine Ethics Committee of the Suzhou Municipal Hospital (ID: K-2021-032-H01).

### Procedure and Data Collection

Serum samples were collected after overnight fasting and analyzed for total cholesterol (TC), high-density lipoprotein cholesterol (HDL-C), low-density lipoprotein cholesterol (LDL-C), very low-density lipoprotein cholesterol (VLDL-C), and triglyceride (TG) levels using Beckman-Coulter AU5800 (Beckman Coulter, Brea, USA) according to the manufacturer's instructions.

All patients received pretest counseling and were given informed consent by genetic counselors before testing for NIPT. Genetic counseling primarily included topics on elderly pregnancy, previous pregnancy affected by fetal aneuploidy, and ultrasonic detection for aneuploidy or fetal abnormalities. The pregnant women screened using NIPT underwent ultrasound before 14 weeks of gestation to determine the number of fetuses, chorionicity, GA, and maternal and fetal characteristics. Medical history including the maternal age, weight, BMI, number of pregnancies, singleton or twin pregnancies, GA, and the method of conception were also recorded.

Blood samples (10 mL) were collected on-site from pregnant women who gave their consent following pretest counseling provided by genetic counselors. cfDNA was extracted using the QIAamp DSP DNA Blood Mini Kit (Qiagen) according to the manufacturer's instructions. The library was constructed using polymerase chain reaction. cfDNA and library concentrations were measured using the QubitTM dsDNA HS Kit (Invitrogen, Carlsbad, CA, USA). Sequencing libraries were then sequenced using the Ion Proton system or the BGISEQ-500 (MGI, China) system. The fetal fraction was evaluated by calculating the proportion of chromosome Y reads. The fetal fraction was reported to be >4%. In samples with a fetal fraction <4%, the laboratory did not generate a risk assessment. All samples had a fetal karyotype (pregnancy with a positive NIPT result) or clinical follow-up results.

### Statistical Analysis

Descriptive data are presented as median and interquartile range for non-normally continuous variables and as absolute values and percentages for categorical variables. The square root (√) of the measured fetal fraction was obtained to make the distribution a Gaussian normality to be assessed using a probability plot. Univariate analysis and multivariate linear regression models were used to examine the associations of the fetal fraction with TG, TC, HDL-C, LDL-C, VLDL-C, maternal weight (kg), GA (weeks), and interval between two tests (weeks). Variance inflation factor (VIF) was used to evaluate multicollinearity; VIF values ≥ 4 require further investigation, whereas VIF values > 10 indicate severe multicollinearity and need to be corrected. Maternal weight and TG levels were highly correlated, and the relationship between GA and the time interval between the two tests was also the same; weight and gestational week were not included in the multiple linear regression model analysis. TG levels were categorized as ≤ 1.10, 1.11–1.70, 1.71–2.30, and >2.30 mmol/L. We also computed estimates and 95% confidence intervals (CIs) for the mean differences in fetal fraction for each category of TG levels. A total of three models were used. Model 1 was a univariate linear regression of the relationship between the TG level and fetal fraction. Model 2 was adjusted for HDL-C and LDL-C levels. In model 3, the time interval between the two tests (weeks) was added on the basis of model 2.

Multiple logistic regression was used to determine the associations of TG, HDL-C, LDL-C and the time interval between the two tests (weeks) with the test failure rate. SPSS version 26.0 (IBM Corp, Armonk, NY, USA) was used for the data analysis. All *P*-values were two-sided, and statistical significance was set at *P* < 0.05.

## Results

### Sample Characteristics

**Figure 1:**
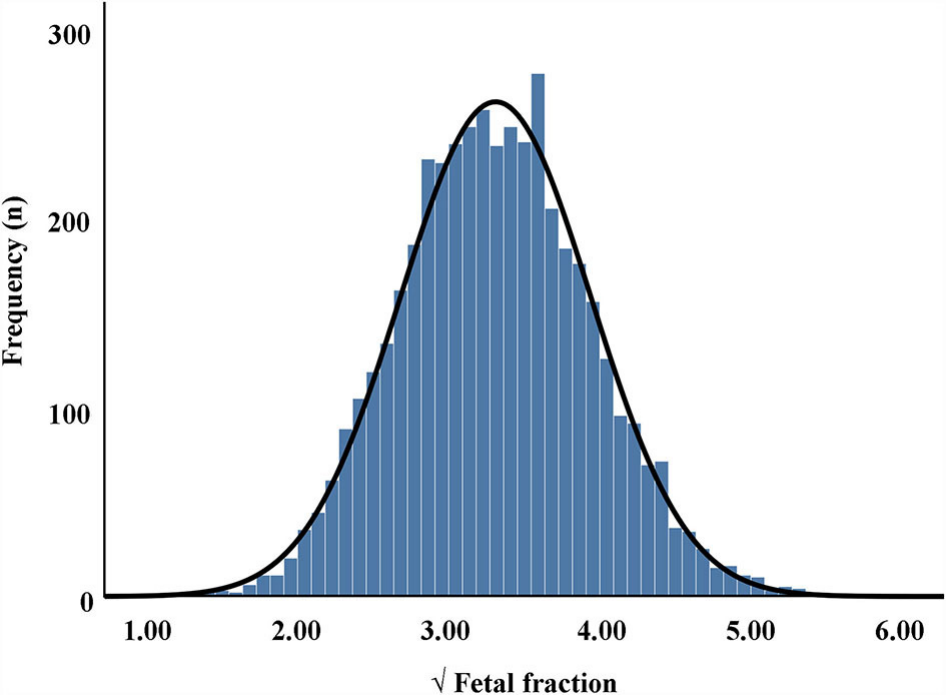
Frequency distribution of square root (√) of fetal fraction in maternal plasma cell-free DNA.

The maternal characteristics of the study population are presented in Table 1, while the frequency distribution of the fetal fraction is shown in Figure 1. The results from 4,514 pregnancies with male fetuses showed that the mean maternal age, weight, NIPT sampling GA, time interval between two tests (weeks), TG, TC, HDL-C, LDL-C, and VLDL-C levels, and fetal DNA concentration were 31 years (28–35), 58 kg (53–63.5), 17 weeks (16–18), 4 weeks (2.43–5.14), 1.42 mmol/L (1.10–1.85), 4.68 mmol/L (4.21–5.22), 1.76 mmol/L (1.54–2.01), 2.46 mmol/L (2.08–2.96), 0.43 mmol/L (0.29–0.56), and 10.88% (8.28–13.89), respectively.

**Table 1:** Sample characteristics of the study population (*n* = 4,514).

**Characteristic**	**Value (median and interquartile range)**
Maternal age (years)	31 (28–35)
Maternal weight (kg)	58 (53–63.5)
Gestational age (weeks)	17 (16–18)
Interval between two tests (weeks)	4 (2.43–5.14)
Fetal fraction (%)	10.88 (8.28–13.89)
TG (mmol/L)	1.42 (1.10–1.85)
TC (mmol/L)	4.68 (4.21–5.22)
HDL-C (mmol/L)	1.76 (1.54–2.01)
LDL-C (mmol/L)	2.46 (2.08–2.96)
VLDL-C (mmol/L)	0.43 (0.29–0.56)

*TG, triglyceride; TC, total cholesterol; HDL-C, high density lipoprotein cholesterol; LDL-C, low density lipoprotein cholesterol; VLDL-C, very low density lipoprotein cholesterol*.

### Relationship Between Lipid Metabolism and Fetal Fraction

Univariate regression analysis demonstrated that the fetal fraction was negatively correlated with maternal weight (regression coefficient = −0.024, *P* < 0.001), TG (regression coefficient = −0.135, *P* < 0.001), TC (regression coefficient = −0.032, *P* = 0.008), LDL-C (regression coefficient = −0.065, *P* < 0.001), and VLDL-C (regression coefficient = −0.195, *P* < 0.001) levels and positively correlated with GA (regression coefficient = 0.034, *P* < 0.001), time interval between the two tests (regression coefficient = 0.021, *P* < 0.001), and HDL-C (regression coefficient = 0.129, *P* < 0.001; Table 2) level. Since the maternal weight was related to the TG level (*r* = 0.221, *P* < 0.001), it was not included in the multiple linear regression analysis. NIPT sampling GA and the interval between the two tests were highly correlated (*r* = 0.697, *P* < 0.001). It is still under consideration whether lengthening the interval will change the effect of lipid metabolism on NIPT. Therefore, only the time interval between the two tests was included in the multiple linear regression study.

**Table 2:** Regression analysis of factors from maternal characteristics and lipid metabolism for predicting √fetal fraction in 4,514 pregnancies with male fetuses.

	**Univariable**		**Multivariable**		
**Independent variable**	**Regression coefficient (95% CI)**	***P***	**Regression coefficient (95% CI)**	**Standardized coefficients**	***P***
Maternal weight (kg)	−0.024 (−0.026 to −0.022)	<0.001	–	–	–
Gestational age (weeks)	0.034 (0.024 to 0.044)	<0.001	–	–	–
Interval between two tests (weeks)	0.021 (0.012 to 0.033)	<0.001	0.016 (0.007 to 0.024)	0.053	<0.001
TG	−0.135 (−0.162 to −0.107)	<0.001	−0.104 (−0.134 to −0.074)	−0.11	<0.001
TC	−0.032 (−0.055 to −0.008)	0.008	–	–	–
HDL-C	0.129 (0.076 to 0.182)	<0.001	0.108 (0.053 to 0.163)	0.059	<0.001
LDL-C	−0.065 (−0.093 to −0.036)	<0.001	−0.036 (−0.066 to −0.006)	−0.037	0.019
VLDL-C	−0.195 (−0.288 to −0.103)	<0.001	–	–	–

*TG, triglyceride; TC, total cholesterol; HDL-C, high density lipoprotein cholesterol; LDL-C, low density lipoprotein cholesterol; VLDL-C, very low density lipoprotein cholesterol*.

Moreover, multivariable regression analysis demonstrated that the fetal fraction was negatively correlated with the TG (standardized coefficients = −0.11, *P* < 0.001) and LDL-C (standardized coefficients = −0.037, *P* = 0.019) levels and positively correlated with the time interval between the two tests (standardized coefficients = 0.053, *P* < 0.001) and the HDL-C (standardized coefficients = 0.059, *P* < 0.001; Table 2) level.

### Association Between TG and Fetal Fraction

**Figure 2:**
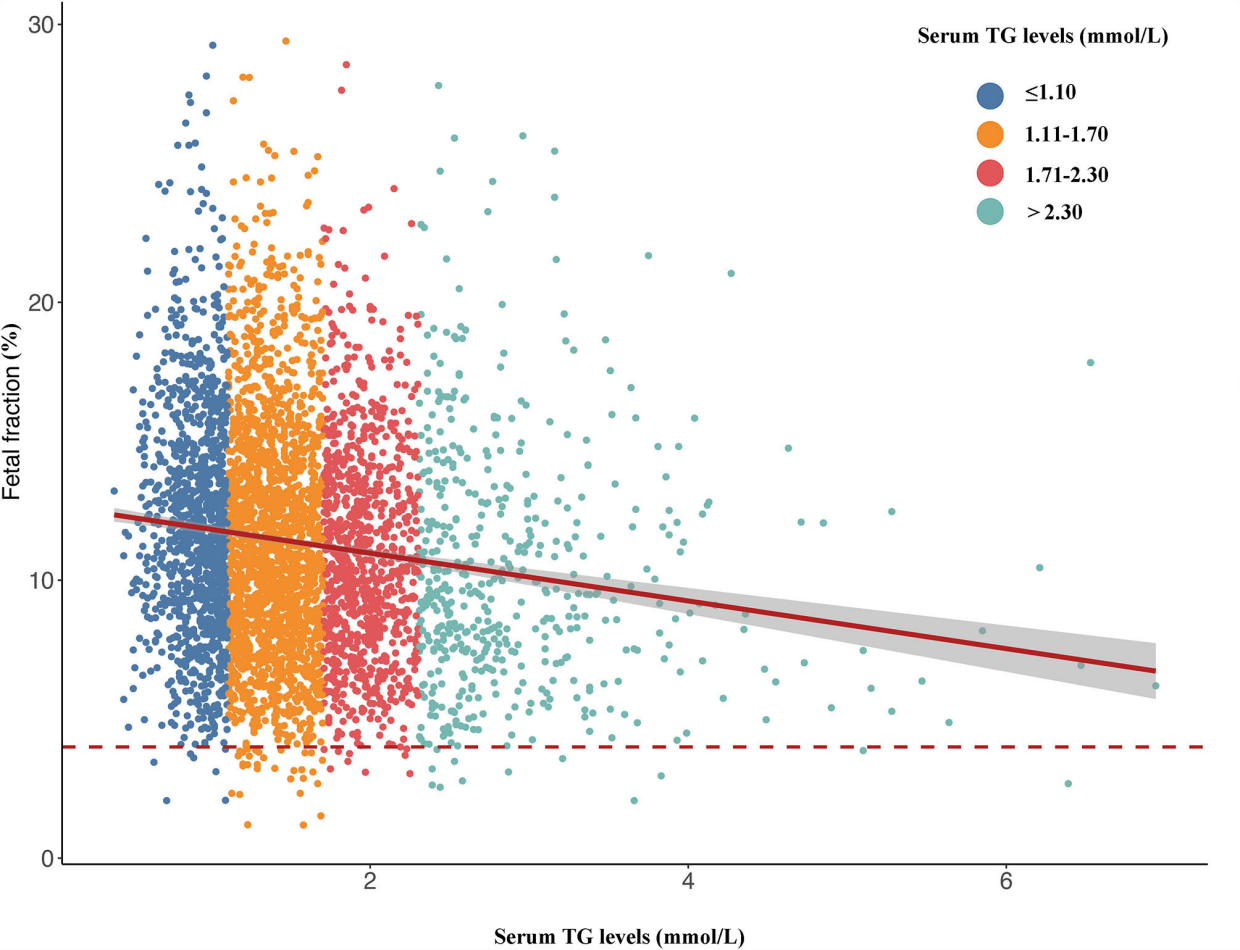
Scatterplots of fetal fraction according to different groups of TG levels with lines of best fit from the linear regression.

**Figure 3:**
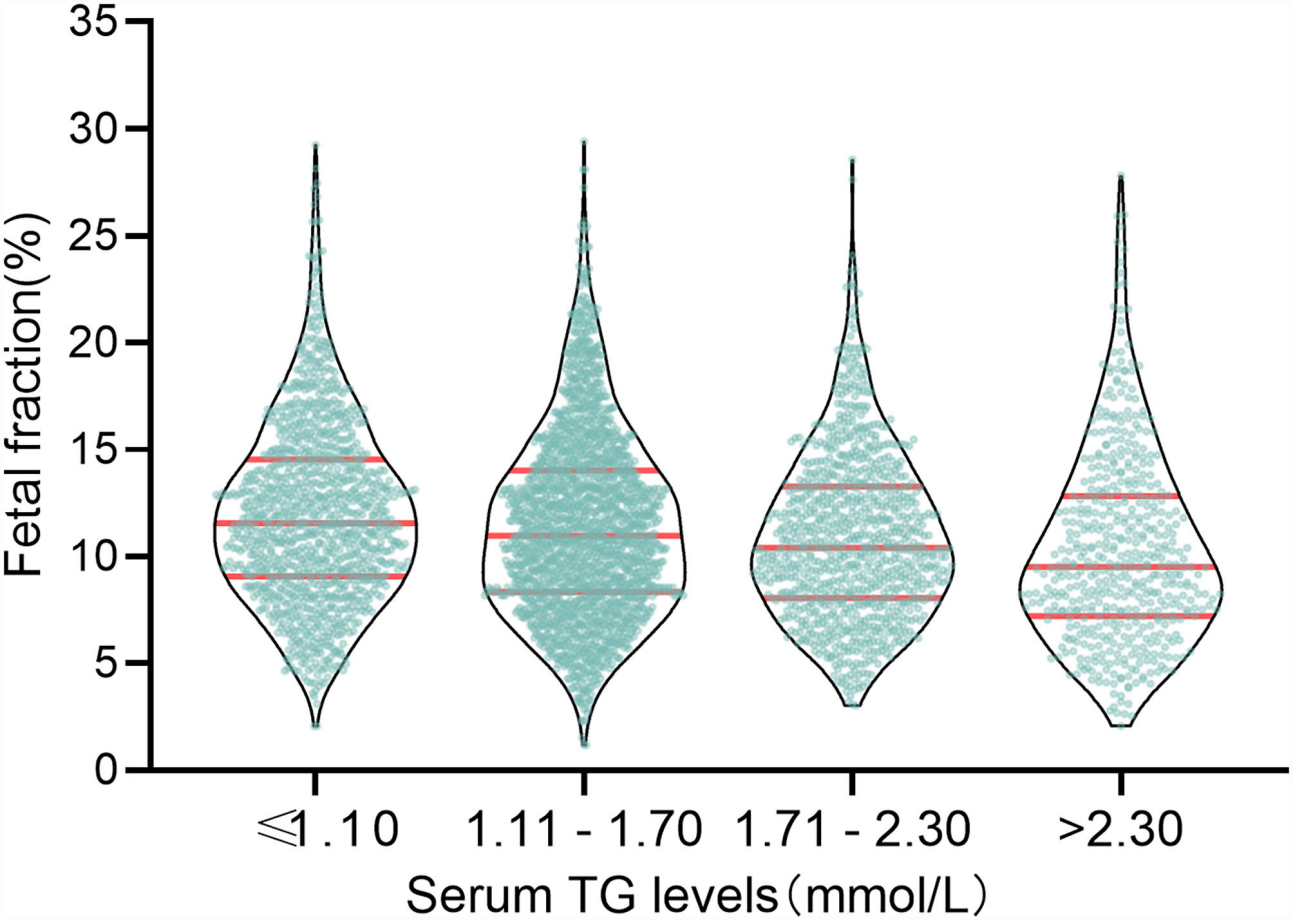
Mean fetal fraction across TG categories.

The above results verify that the TG level was negatively correlated with fetal fraction in NIPT (Figure 2). TG levels were classified into four categories: ≤ 1.10, 1.11–1.70, 1.71–2.30, and >2.30 mmol/L. The numbers of pregnant women with TG levels in these four categories were 1163, 1923, 930, and 498, respectively. The mean fetal fractions across the TG categories were 11.56% (9.06–14.53), 10.96% (6.33–14.02), 10.41% (8.05–13.27), and 9.51% (7.22– 12.81), respectively (Figure 3).

**Figure 4:**
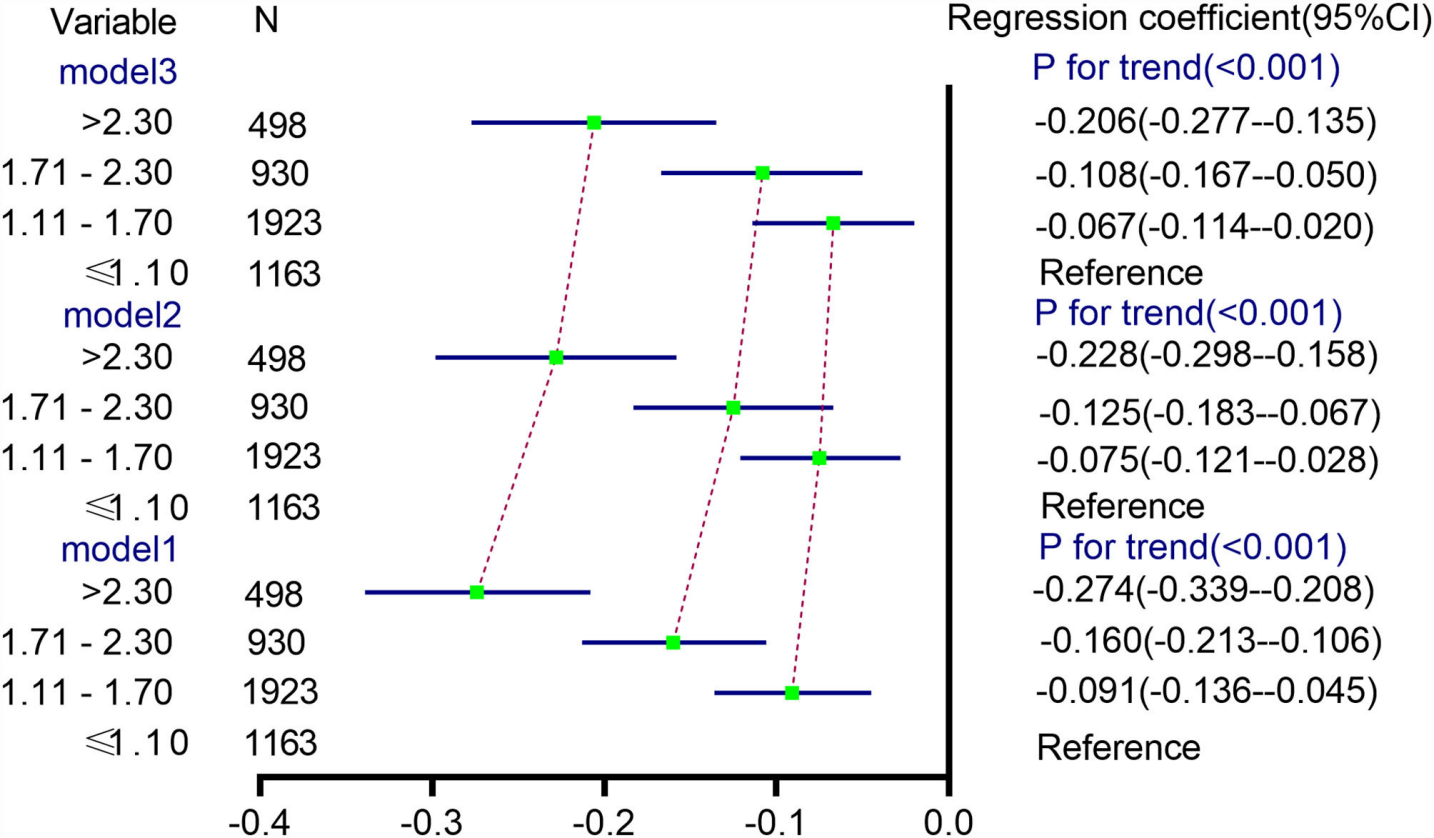
Differences in √fetal fraction according to TG categories. Model 1 was a univariate linear regression of the relationship between the TG level and √fetal fraction. Model 2 was adjusted for HDL-C and LDL-C levels. In model 3, the time interval between two tests (weeks) was added on the basis of model 2.

The multivariable-adjusted (model 2) mean √fetal fraction differences across the categories of TG levels were −0.075 (95% CI: −0.121 to −0.028) for TG levels of 1.11–1.70 mmol/L, −0.125 (95% CI: −0.183 to −0.067) for TG levels of 1.71–2.30 mmol/L, and −0.228 (95% CI: −0.298 to −0.158) for TG levels > 2.30 mmol/L compared with TG levels ≤ 1.10 mmol/L (P_*trend*_ < 0.0001). Nevertheless, the mean √fetal fraction differences were −0.067 (95% CI: −0.114 to −0.020) for TG levels of 1.11–1.70 mmol/L, −0.108 (95% CI: −0.167 to −0.050) for TG levels of 1.71–2.30 mmol/L, and −0.206 (95% CI: −0.277 to −0.135) for TG levels > 2.30 mmol/L compared with TG levels ≤ 1.10 mmol/L (P_*trend*_ < 0.0001) after adjusting for confounding factors plus the time interval between two tests (weeks) (model 3, Figure 4), suggesting that delaying the time of NIPT blood collection significantly decreases the mean fetal fraction differences between the different TG groups, especially in pregnant women with elevated TG levels by about 10%.

### Association Between TG and Test Failure Rate of NIPT

**Figure 5:**
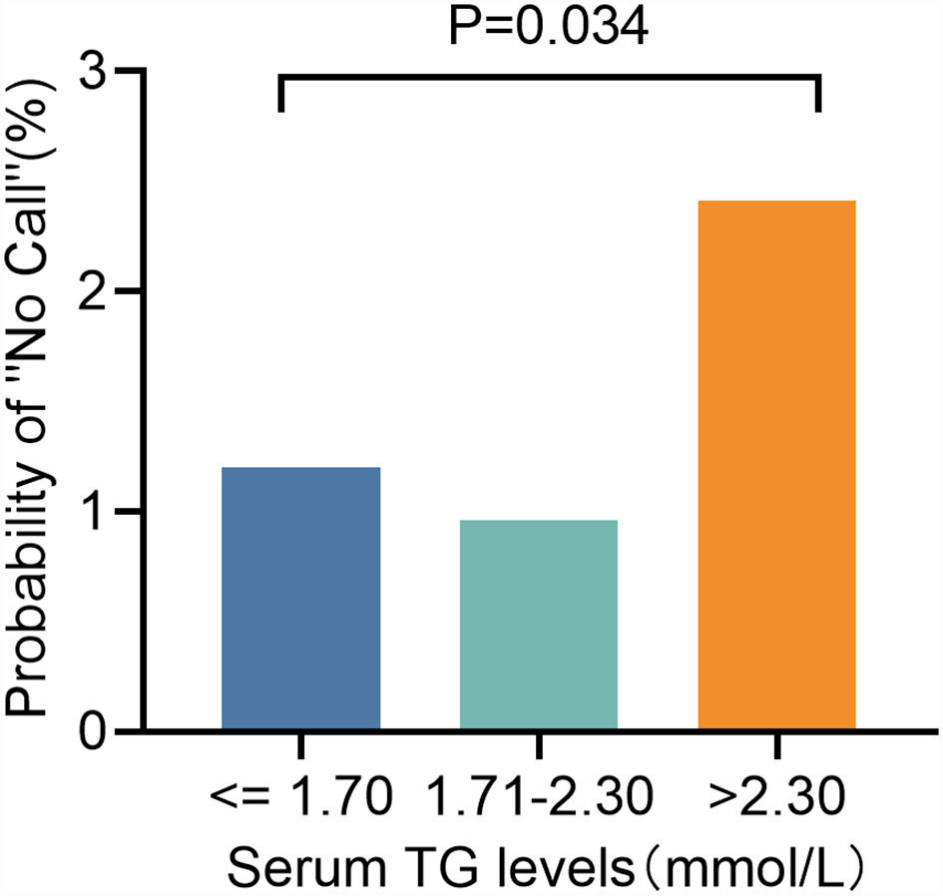
Test failure rate according to different groups of TG.

In the logistic regression analyses, augmented TG [odds ratio (OR): 1.560, 95% CI: 1.177–2.068, *P* = 0.002; Table 3] levels were significant predictors of test failure. We then classified TG levels into the following three categories: ≤1. 70, 1.71–2.30, and >2.30 mmol/L. Although they are still low, the rate of screen failures increased with TG levels from 1.20% at ≤1.70 mmol/L to 2.41% (*P* = 0.034) at >2.30 mmol/L (2.41%, *P* = 0.034) (Figure 5).

**Table 3:** Logistic regression analyses demonstrating factors that contribute to test failure rate.

	**Univariable**	
**Independent variable**	**Odds ratio (95% CI)**	***P***
Interval between two tests (weeks)	0.969 (0.857–1.096)	0.619
TG	1.560 (1.177–2.068)	0.002
TC	0.956 (0.685–1.336)	0.794
HDL-C	0.845 (0.397–1.800)	0.663
LDL-C	0.908 (0.603–1.366)	0.643
VLDL-C	2.211 (0.629–7.767)	0.216

## Discussion

Accurate identification of fetal aneuploidy depends on sufficient fetal fraction using the NIPT method. To date, several strategies have been used to increase the relative abundance of fetal cfDNA and reduce the failure rate of NIPT (14, 20–22). Welker et al. (22) performed a new NIPT method that sequenced shorter cfDNA fragments (<140 bp) to significantly improve the fetal fraction, with an average fetal fraction increase of 2.3-fold. Moreover, the zero sample had a fetal fraction < 4% when screened with fetal fraction amplification, whereas 3.7% of the same patients had fetal fraction < 4% without fetal fraction amplification. However, the original fetal fraction information may be lost when using this new technology, and further studies are needed to evaluate this. Studies on factors affecting fetal fraction aim to increase the relative abundance of fetal cfDNA by regulating these factors, which may reduce the test failure rate. To the best of our knowledge, this is the largest and most extensive study to determine the associations of lipid metabolism with the fetal fraction and test failure rate in the NIPT method. Among the 4,514 pregnancies with male fetuses, TG, which was a significant factor (standardized coefficient: −0.11), and LDL-C levels were negatively correlated with fetal fraction. Conversely, the HDL-C level and the time interval between the two tests were positively correlated with fetal fraction. Hence, laboratories should be aware that excessive TG concentrations will reduce fetal-derived cfDNA and affect the accuracy of NIPT in screening aneuploidy.

Our results suggested that the fetal fraction was negatively associated with TG and LDL-C levels and positively correlated with the HDL-C level. In the logistic regression analyses, we found that an augmented TG level (OR: 1.560, 95% CI: 1.177–2.068, *P* = 0.002; Table 3) was a significant predictor of test failure. After classifying the TG levels, we found that the rate of screen failures increased with an increase in TG. Additionally, complex changes occurred in the lipid profiles during pregnancy. Brizzi et al. (23) investigated the changes in lipoproteins and lipids in women during normal pregnancy and compared their results with those obtained in nulliparous women with similar age. Pregnant women had increased TG levels vs. nulliparous and primiparous women (24). Interestingly, in the MESA (25), NHANES (26) and UK Biobank studies (27) TG demonstrated the strongest positive association with leukocyte count. Hu et al. (28) also found that peripheral leukocyte counts are extensively associated with serum lipid levels, with patterns differing by leukocyte subsets. In view of the close relationship between TG and leukocyte counts, the data indicate that TG may be directly involved in leukogenesis. cfDNA in the peripheral blood sample of a pregnant woman is derived from three tissues: the placenta, maternal bone marrow, and fetus. Moreover, cfDNA derived from the white-cell lineage typically contributes to more than 70%, sometimes even to up to 90% (29, 30). As such, we speculate that lipids during pregnancy, especially TG levels, affect the fetal fraction by participating in leukogenesis. At present, there are limited reports on the relationship between TG and low fetal fraction. Our study provides a starting point for more in-depth exploration of the impact of lipid metabolism on NIPT in the future.

The positive association between the time interval between the two tests and fetal fraction is compatible with a previous study (31). Although the fetal fraction changes slowly with GA, the study indicates that by 8–9 days after the initial draw, there is sufficient cfDNA to expect a 55–60% chance of success. In this study, our results indicated that delaying the time of NIPT blood collection significantly decreased (about 10%) the mean fetal fraction differences between the TG groups, especially in pregnant women with elevated TG (>2.30 mmol/L) levels. It is more likely that apoptotic placental cells (trophoblasts) are a major source of fetal-derived cfDNA in maternal plasma (32), and the number of apoptotic placental cells is proportional to the mass. Therefore, as the interval increases, the weight of the placenta increases and its apoptotic release increases. However, the test failure differences between the TG groups were not influenced by the time interval between the two tests. This may be because there are fewer failed samples which can affect the statistical performance. In addition, due to limitations within our clinical data, the following considerations should be noted: First, the relationship between lipid metabolism and fetal fraction was studied according to data obtained at from a single center. Larger samples are recommended for further evaluation. Second, although we adjusted for many contributing variables, such as biochemical placental markers (free β-hCG, PAPP-A, and PlGF) and *in vitro* fertilization conception (33), other relevant variables may have been overlooked.

## Conclusion

In summary, our results show that TG and LDL-C levels were negatively correlated with fetal fraction, while HDL-C level and the time interval between the two tests were positively correlated with fetal fraction. Lower TG level and delay in the time between NIPT blood collections may be more conducive to obtaining sufficient fetal fractions, thereby providing a way to effectively attenuate test failures and further improve test analytical performance.

## Data Availability Statement

The datasets for this article are not publicly available to assure patient confidentiality and participant privacy. Requests to access the datasets should be directed to Ting Wang, biowt@njmu.edu.cn.

## Ethics Statement

Written informed consent was obtained from the individual(s) for the publication of any potentially identifiable images or data included in this article.

## Author Contributions

JC, LQ, JJ, and SZ: conception, design, collection, and assembly of data. PC: administrative support. HT, ZY, and JS: provision of study materials or patients. TW and YL: data analysis and interpretation. All authors: manuscript writing and final approval of manuscript.

## Funding

This study was supported by the National Natural Science Foundation of China (Grant No. 81901632 and 82001576); Suzhou Science and Technology Support Program (SYS2019095, SYS2019098, BE2019683, and SS2019066); Jiangsu Science and Technology Support Program (SBE2019740167); Suzhou Clinical Medical Expert Team (SZYJTD201708); Jiangsu Provincial Medical Innovation Team (CXTDB2017013).

## Conflict of Interest

The authors declare that the research was conducted in the absence of any commercial or financial relationships that could be construed as a potential conflict of interest.

## Publisher's Note

All claims expressed in this article are solely those of the authors and do not necessarily represent those of their affiliated organizations, or those of the publisher, the editors and the reviewers. Any product that may be evaluated in this article, or claim that may be made by its manufacturer, is not guaranteed or endorsed by the publisher.
